# Dietary Omega-3 Fatty Acids from Fish and Risk of Metabolic Dysfunction-Associated Steatotic Liver Disease in a Mediterranean Population: Findings from the NUTRIHEP Cohort

**DOI:** 10.3390/nu17213372

**Published:** 2025-10-27

**Authors:** Rossella Tatoli, Bonfiglio Caterina, Rossella Donghia, Pasqua Letizia Pesole, Luigi Fontana, Gianluigi Giannelli

**Affiliations:** 1 Unit of Data Science, National Institute of Gastroenterology IRCCS “Saverio de Bellis”, Castellana Grotte, 70013 Bari, Italy; catia.bonfiglio@irccsdebellis.it (B.C.); rossella.donghia@irccsdebellis.it (R.D.); 2 Core Facility Biobank, National Institute of Gastroenterology IRCCS “Saverio de Bellis”, Castellana Grotte, 70013 Bari, Italy; letizia.pesole@irccsdebellis.it; 3 Charles Perkins Centre, Faculty of Medicine and Health, University of Sydney, Sydney, NSW 2006, Australia; luigi.fontana@sydney.edu.au; 4 Department of Endocrinology, Royal Prince Alfred Hospital, Sydney, NSW 2050, Australia; 5 Scientific Direction, National Institute of Gastroenterology IRCCS “Saverio de Bellis”, Castellana Grotte, 70013 Bari, Italy; gianluigi.giannelli@irccsdebellis.it

**Keywords:** MASLD, omega-3 fatty acids, DHA, EPA

## Abstract

**Background**: Metabolic Dysfunction-Associated Steatotic Liver Disease (MASLD) is linked to metabolic syndrome, obesity, and type 2 diabetes. Omega-3 fatty acids, especially EPA and DHA from fish, may protect against hepatic steatosis. **Methods**: From 2015 to 2018, all participants were invited to the first follow-up, where 1426 (62% response rate) responded and underwent the same standardized protocol as at baseline. For this analysis, the study is a cross-sectional investigation focusing solely on follow-up data, which included 1297 adults. MASLD was diagnosed via standardized ultrasound, and dietary intake was assessed using the validated EPIC Food Frequency Questionnaire. Associations between total EPA and DHA intake and MASLD were examined using logistic regression models adjusted for age, sex, marital status, occupation, income, Mediterranean diet adherence, liver enzymes, and C-reactive protein. **Results**: Overall, 48.5% of participants had MASLD. Higher total EPA and DHA intake was associated with lower odds of MASLD (EPA highest vs. lowest quartile: OR = 0.572, 95% CI 0.400–0.818; DHA highest vs. lowest quartile: OR = 0.516, 95% CI 0.361–0.739). Intake of fatty fish contributed most strongly to this protective effect (EPA highest vs. lowest quartile: OR = 0.556, 95% CI 0.390–0.794; DHA highest vs. lowest quartile: OR = 0.575, 95% CI 0.403–0.820), whereas mollusks, crustaceans, and some processed/frozen fish showed weaker or no associations. A statistically significant trend of decreasing MASLD risk was observed across increasing quartiles of both EPA and DHA intake. **Conclusions**: Higher intake of EPA and DHA, especially from fatty fish, is linked to lower MASLD risk in this Mediterranean population, supporting recommendations for regular fish consumption to protect liver health.

## 1. Introduction

Metabolic Dysfunction-Associated Steatotic Liver Disease (MASLD) has emerged as one of the most prevalent chronic non-communicable diseases worldwide, affecting nearly one-third of the adult population [1,2,3,4]. In Italy, MASLD presents as an epidemiologically significant problem, with estimates suggesting that between 20% and 40% of the general population (on average 1 in 2–5 Italians) suffers from steatosis. These figures are even more pronounced in obese and diabetic patients, where prevalence rates may soar to between 50% and 90% [5]. The term MASLD was recently introduced to define steatotic liver disease associated with metabolic syndrome [3,4], as outlined in a multi-society Delphi consensus statement from 2023. This new terminology replaces the term NAFLD and emphasizes the close connection between fatty liver and metabolic syndrome.

Closely linked with visceral obesity, type 2 diabetes, and other components of metabolic syndrome, MASLD is now a leading cause of liver-related morbidity, mortality, and transplantation [3]. Previous research has attempted to test a treatment for fatty liver disease [6]; however, the most effective approach continues to be lifestyle changes.

Its growing prevalence highlights the urgent need to better understand modifiable risk factors and potential protective dietary components.

MASLD has a multifactorial pathophysiology, with the liver as the central organ and other tissues contributing to disease progression [7,8]. Key drivers include insulin resistance, dyslipidemia, inflammation, and mitochondrial dysfunction, with additional risk from genetic factors, gut microbiota alterations, and diets high in fat, sugar, and low in fiber [9,10,11,12,13,14]. Among dietary factors, omega-3 polyunsaturated fatty acids (PUFAs), especially eicosapentaenoic acid (EPA) and docosahexaenoic acid (DHA), have been studied for their anti-inflammatory, anti-fibrotic, antioxidant, and insulin-sensitizing properties [15,16,17,18,19]. Mainly derived from fish [20,21], these fatty acids may counterbalance the excessive omega-6 intake typical of Western diets and help reduce hepatic fat, though evidence remains mixed [18].

This study investigates the association between MASLD and the consumption of omega-3 fatty acids derived from fish using data from the NUTRIHEP cohort, a prospective study conducted in a Mediterranean population with traditionally high fish consumption. There are several studies in the literature that have evaluated the short- and long-term effects of omega-3 supplementation on the liver, with conflicting results [6,22,23]. In this study, the focus is deliberately limited to dietary omega-3s derived from fish consumption because lifestyle intervention currently remains the most effective treatment for fatty liver disease [6]. Fish consumption is highly recommended for MASLD prevention, not only for its high-quality protein but also for its content of polyunsaturated fatty acids [24,25].

### Omega-3 Fatty Acids

Omega-3 fatty acids are long-chain polyunsaturated fatty acids (PUFAs), characterized by the presence of a double bond at the third carbon atom of the hydrocarboxyl chain, counting from the methyl end [22]. These fatty acids are synthesized from α-linolenic acid, which is the simplest fatty acid. Both α -linolenic acid and linoleic acid (from which omega-6 fatty acids are derived) are defined as “essential fatty acids” because animal organisms, including humans, cannot independently biosynthesize them and must obtain them through diet. In the liver, dietary α -linolenic acid is metabolized by Δ5 and Δ6 desaturase enzymes to form eicosapentaenoic acid (EPA) and docosahexaenoic acid (DHA).

The concentration of omega-6 fatty acids significantly influences the biological activity of omega-3 fatty acids, as the metabolic pathways of these two groups of PUFAs are interconnected. These two classes of PUFAs have opposing physiological functions and are competitively metabolized by the same enzymatic system (Δ5-Δ6 elongases and desaturases), which exhibits a higher affinity for metabolizing omega-3 than omega-6 PUFAs [26]. Linoleic acid, the precursor of omega-6, is metabolized into arachidonic acid, which gives rise to mediators with pro-inflammatory and pro-thrombotic properties. In contrast, EPA and DHA are involved in the biosynthesis of metabolites that possess anti-inflammatory properties [18]. A healthy diet should maintain a ratio of 4.1 between omega-6 and omega-3 fatty acids [27]. When dietary intake of omega-6 is significantly higher, eicosanoids derived from arachidonic acid dominate over the anti-inflammatory eicosanoids derived from omega-3 fatty acids.

The omega-3 PUFAs play a significant role in liver function. An imbalanced dietary intake of omega-3 and omega-6 fatty acids plays a crucial role in the development of liver steatosis [18].

In the liver, omega-3 fatty acids may exert potential beneficial effects through various mechanisms [19]. They play a role in glucose homeostasis, enhancing insulin sensitivity and preventing the progression of NAFLD [20]. Omega-3 fatty acids can also reduce hepatic steatosis via upregulation of lipid oxidation, and they possess anti-inflammatory, antioxidant, anti-thrombotic, and anti-fibrotic properties [19]. Omega-3 fatty acids downregulate gene expression of several transcriptional factors involved in lipogenesis and triglyceride accumulation in the liver. In particular, they can downregulate gene expression of SREBP-1c by inhibiting activation of the liver X receptor, thereby reducing lipogenesis [28,29,30]. Jump et al. have shown that DHA has a specific role in downregulating gene expression of SREBP-1c, through the 26S proteasomal degradation of nuclear SREBP-1c61 [31].

Omega-3 fatty acids are activators of peroxisome proliferator-activated receptors (PPARs) that act as nuclear transcription factors and regulate the expression of genes involved in lipid, carbohydrate, and protein metabolism [32]. There are three different forms of PPARs: PPARα, PPARβ/δ, and PPARγ. PPARα is mainly expressed in the liver. Omega-3 fatty acids activate PPARα and PPARβ/δ, promoting mitochondrial and peroxisomal fatty acid oxidation and decreasing intrahepatic triglyceride accumulation [33]. Moreover, the activation of PPARγ decreases the production of pro-inflammatory cytokines such as TNF-a and IL-6 that in turn reduces hepatic inflammation [27].

EPA and DHA are the most important fatty acids in the omega-3 group. They are found in a variety of foods, with fish (particularly fatty fish) and other seafood being the richest sources. Since dietary α-linolenic acid conversion alone does not meet daily requirements, EPA and DHA intake through food must be the primary source [20,21]. Additionally, the activity of desaturase enzymes can be influenced by various factors, including diet, obesity, insulin resistance, oxidative stress, and liver disease. The results of Araya et al.’s study indicate that the activity of Δ5 and Δ6 is lower in obese individuals with NAFLD compared to non-obese individuals without NAFLD [34].

A daily intake of EPA and DHA ranging from 0.25 to 2 g is deemed sufficient to meet the needs of the human body [27]. This quantity can be obtained by consuming two portions of fish per week, with at least one portion being oily fish [35].

## 2. Materials and Methods

### 2.1. Study Population

The NUTRIHEP cohort study began in 2005–2006, selecting a systematic random sample of individuals aged 18 or older from the patient list of primary care physicians in Putignano (BA), Italy [36]. From 2015 to 2018, all participants were invited to the first follow-up, where 1426 (62% response rate) responded and underwent the same standardized protocol as at baseline [37]. Written informed consent was obtained from all participants after providing detailed information about the use of medical data. For this analysis, the study is a cross-sectional investigation focusing solely on follow-up data. The Ethical Committee of the Minister of Health approved the study (DDG-CE-792/2014) on 14 February 2014.

### 2.2. Data Collection

During follow-up visits, participants underwent all assessments outlined in the study protocol. Trained physicians and/or nutritionists carried out structured interviews to gather sociodemographic, medical, and lifestyle data, such as smoking history, dietary habits, education level, occupation, and marital status.

Anthropometric measurements were taken with participants wearing only underwear and no shoes. Body weight was measured to the nearest 1 kg using an electronic scale (SECA©, Hamburg, Germany), and height to the nearest 1 cm with a wall-mounted stadiometer (SECA©). Blood pressure (BP) was measured following international guidelines [38,39], and the average of three readings was calculated. Dietary habits were assessed using the validated European Prospective Investigation into Cancer and Nutrition (EPIC) Food Frequency Questionnaire (FFQ) [40,41], completed independently by participants and verified by nutritionists before being processed with a dedicated online tool to determine micro- and macronutrient intake.

Biochemical analyses included fasting serum glucose (FSG) and insulin, HbA1c, triglycerides, total cholesterol, LDL-C, HDL-C, AST, ALT, ALP, GGT, ferritin, and high-sensitivity C-reactive protein. Measurements were performed using the COBAS 8000 autoanalyzer (ROCHE Diagnostics SPA, Monza, Italy). Insulin resistance was estimated using the Homeostasis Model Assessment of Insulin Resistance (HOMA-IR) [42], calculated as follows:

HOMA-IR = FSG (mg/dL) × fasting Insulin (μIU/mL)/405.

Liver steatosis was evaluated using a standardized ultrasound exam (Hitachi H21 Vision, Hitachi Medical Corporation, Tokyo, Japan) with a 3.5 MHz transducer. Hepatic fat content was semi-quantitatively scored based on liver echotexture, echo penetration, visibility of intrahepatic vessels, and diaphragm differentiation [43]. Figure S1 shows the ultrasound reference chart used for steatosis grading.

### 2.3. Outcome Assessment

**Figure 1:**
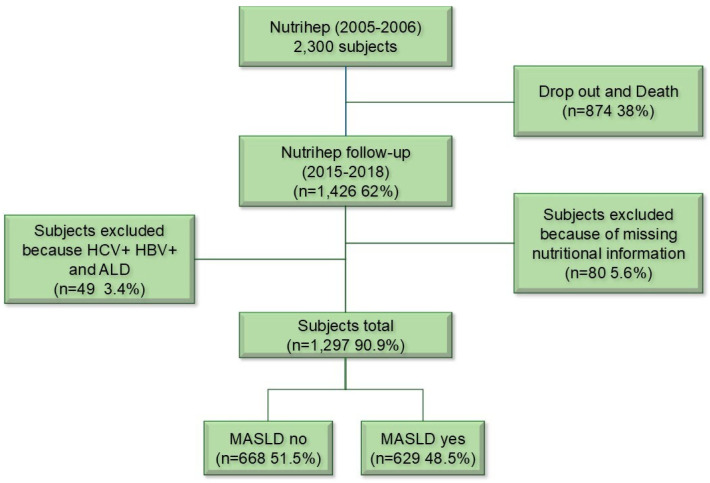
Flow Chart. ALD: alcohol-related liver disease; MASLD: Metabolic Dysfunction-Associated Steatotic Liver Disease.

MASLD is characterized by hepatic steatosis along with at least one cardiometabolic risk factor: (1) BMI over 25 kg/m^2^ or waist circumference exceeding 94 cm in men and 80 cm in women; (2) fasting glucose level of 100 mg/dL or higher, 2-h post-load glucose of 140 mg/dL or higher, HbA1c at or above 5.7%, or use of glucose-lowering medication; (3) blood pressure of 130/85 mmHg or higher, or currently on antihypertensive treatment; (4) triglyceride levels of 150 mg/dL or above, or on lipid-lowering therapy; (5) HDL cholesterol below 40 mg/dL in men and below 50 mg/dL in women, or targeted lipid-lowering therapy. In line with previous NAFLD criteria, the MASLD diagnosis also required limited alcohol intake—less than 20–50 g/day for women and less than 30–60 g/day for men [4]. To prevent confounding, individuals with other liver diseases (such as co-infection with HCV or HBV in MASLD) were excluded from the analysis [3] (see Figure 1).

### 2.4. Exposure Variable

Intake of EPA and DHA was estimated from participants’ reported fish consumption using EPIC FFQ. Nutrient values for EPA and DHA were obtained from the Food Composition Database for Epidemiological Studies in Italy (BDA; https://bda.ieo.it/?page_id=690&lang=en, accessed on 2 September 2025), a comprehensive national reference on food composition. Reported intakes were expressed as total daily EPA and DHA and categorized into three groups, as summarized in Table 1.

### 2.5. Confounding Variables

Covariates were chosen based on previous research and clinical or statistical considerations for their potential link to MASLD. After checking for collinearity, we included demographic and lifestyle variables such as age, gender, occupation, marital status, personal income, and adherence to the Relative Mediterranean Diet (rMED), along with lab measurements like AST/ALT, GGT, ALP, and C-reactive protein. Variables used to define MASLD (BMI, waist circumference, fasting glucose, triglycerides, blood pressure, HDL cholesterol, and HbA1c) were excluded to prevent collinearity.

### 2.6. Statistical Analysis

Differences between groups were assessed using the Wilcoxon test for continuous variables and the χ^2^ test for categorical variables. Logistic regression models were used to estimate odds ratios (ORs) and 95% confidence intervals (CIs), with MASLD as the outcome and EPA and DHA intake (both as total daily intake and categorized into three groups) as predictors. Intakes were analyzed as continuous variables and by quartiles. ORs indicate the association between exposure and outcome: OR = 1 indicates no association, OR >1 indicates increased risk, and OR < 1 indicates a protective effect [44].

Two models were constructed: Model 1 unadjusted, and Model 2 adjusted for age (<40 vs. ≥40 years), sex, marital status, occupation, self-reported family income, rMED score, ALP, γGT, AST/ALT, and C-reactive protein. Candidate confounders were initially selected based on the literature and then refined using the Least Absolute Shrinkage and Selection Operator (LASSO) method [45]. Variables defining MASLD (BMI, waist circumference, HDL cholesterol, triglycerides, fasting glucose, HbA1c, blood pressure) were excluded to avoid collinearity, and the Variance Inflation Factor (VIF) was used to detect multicollinearity, with variables removed if VIF > 5 [46]. A forest plot was generated to compare ORs for EPA and DHA intake quartiles in multivariate models.

Continuous variables were summarized as means ± standard deviations (M ± SD) or medians with interquartile ranges, while categorical variables were presented as frequencies and percentages. Two-tailed significance was set at *p* < 0.05 to test the null hypothesis of non-association. Analyses were performed using Stata 19 (StataCorp 2025, College Station, TX, USA) and Rstudio (“Mariposa Orchid” release).

## 3. Results

### 3.1. Participant Characteristics

Table 2 summarizes the characteristics of 1297 participants, stratified by MASLD status. Overall, 629 participants (48.50%) had MASLD, including 44.00% of women (n = 327/744) and 54.60% of men (n = 302/553). Participants with MASLD were generally older and had a higher prevalence of hypertension and hyperlipidemia. They also showed higher BMI (mean 30.28 ± 4.97 kg/m^2^) and weight (mean 79.58 ± 14.73 kg) compared to those without MASLD. Educational attainment was lower in the MASLD group, with 423 individuals having primary or secondary education versus 242 in the non-MASLD group, and only 59 MASLD participants were university graduates compared to 119 in the non-MASLD group. The evaluation of family income indicates that people without MASLD are generally in a better financial position than those with MASLD: a total of 56 individuals (66.70%) reported having enough money available, compared to 28 (33.30%). Most of our sample (1019 subjects) claim to have sufficient family income.

Blood parameters were elevated in MASLD participants, with significant differences observed for multiple markers.

Notable differences were observed in omega-3 intake, with MASLD subjects consuming less EPA (134.11 mg/day) and less DHA (232.99 mg/day). These differences in EPA and DHA intake were present across the three groups, but statistical significance was only reached in the third group (*p*-value = 0.027 for EPA and 0.022 for DHA).

Table 3 presents a breakdown of the EPA content in fish based on MASLD. The analysis indicates that there are no statistically significant differences in intake between individuals with MASLD and those without, except for EPA intake from sardines, mackerel, anchovies (*p*-value 0.010), salmon (*p*-value < 0.001), trout (*p*-value 0.034), swordfish, tuna (*p*-value 0.016), salted cod, stockfish (*p*-value 0.001), and canned fish (*p*-value 0.011).

Table 3 also presents a breakdown of the DHA content in fish based on MASLD. There are no statistically significant differences in intake between those with MASLD and those without, except for DHA intake from sardines, mackerel, anchovies (*p*-value 0.010), salmon (*p*-value < 0.001), trout (*p*-value 0.034), swordfish, tuna (*p*-value 0.016), salted cod, stockfish (*p*-value 0.001), and canned fish (*p*-value 0.011).

### 3.2. Associations Between Daily EPA and DHA Intake and MASLD

To begin, we performed a univariate logistic regression analysis to examine the relationship between MASLD and the intake of EPA and DHA.

Table 4 shows the results of logistic regression models evaluating the association between MASLD and total EPA and DHA intake, analyzed both as continuous variables and categorized into intake groups.

The multivariate logistic regression results in Table 4 show that the lowest odds ratio (OR) for total EPA intake was observed in the 183–630 mg/day quartile [OR 0.572, 95% CI 0.400; 0.818, *p*-value = 0.002] compared to <77 mg/day, after adjusting for covariates. This indicates that individuals in the fourth quartile had a 47.2% lower likelihood of developing MASLD, after adjusting for age (<40 vs. ≥40 years), gender, marital status, occupation, family income, rMED, ALP, γGT, AST/ALT, and C-reactive protein.

The lowest OR for total DHA intake was observed in the 319–950 mg/day quartile [OR 0.516, 95% CI 0.361–0.739, *p* < 0.001] compared to less than 130 mg/day, indicating a 41.6% lower likelihood of MASLD in the multivariate model (Table 4).

Analysis of EPA and DHA as continuous variables showed a modest negative association with MASLD risk (EPA: OR = 0.999, 95% CI 0.997–0.999, *p* = 0.048; DHA: OR = 0.999, 95% CI 0.998–1.000, *p* = 0.061), suggesting that higher omega-3 intake may slightly lower MASLD risk. A statistically significant trend of decreasing MASLD risk was observed across increasing quartiles of both EPA and DHA intake (Table 4).

**Figure 2:**
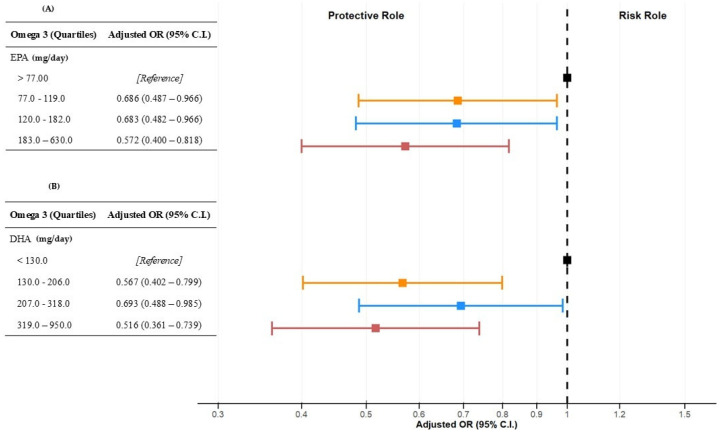
Forest plot of odds ratios (ORs) and 95% confidence intervals (CIs) for MASLD across EPA (**A**) and DHA (**B**) intake quartiles (mg/day). **Abbreviations:** MASLD: Metabolic Dysfunction-Associated Steatotic Liver Disease; OR: odds ratio; CI: confidence interval.

Figure 2 presents a forest plot of ORs and 95% confidence intervals for EPA and DHA quartiles.

Table 5 shows logistic regression results examining the association between MASLD and EPA and DHA intake from Group 1, analyzed as continuous and categorical variables. For EPA, in Model 2, the lowest odds ratio (OR) was observed in the 11.80–23.84 mg/day quartile [OR 0.667, 95% CI 0.476–0.936, *p* = 0.019] compared to <4.14 mg/day, after adjusting for covariates. In the highest quartile (23.85–198.0 mg/day), the OR was 0.679 [95% CI 0.483–0.955, *p* = 0.026]. For DHA, the lowest OR was in the 7.82–16.99 mg/day quartile [OR 0.647, 95% CI 0.461–0.908, *p* = 0.012] versus <2.80 mg/day, after adjusting for covariates. In the fourth quartile (17.0–143.3 mg/day), the OR was 0.684 [95% CI 0.487–0.961, *p* = 0.029]. No statistically significant associations were observed for total EPA or DHA intake from Group 1 fish consumption.

Table 6 presents the logistic regression results examining the association between MASLD and EPA and DHA intake from Group 2, analyzed as both continuous and categorical variables.

Analyzing the multivariate model, for EPA, the lowest odds ratio (OR) was observed in the 110.3–470.0 mg/day quartile [OR 0.556, 95% CI 0.390–0.794, *p* = 0.001] compared to <29.29 mg/day, after adjusting for covariates. In the third quartile (64.01–110.2 mg/day), the OR was 0.678 [95% CI 0.479–0.960, *p* = 0.028]. For DHA, the lowest OR was found in the 204.8–800.0 mg/day quartile [OR 0.575, 95% CI 0.403–0.820, *p* = 0.002] versus <54.6 mg/day, after adjusting for covariates. In the third quartile (118.5–204.7 mg/day), the OR was 0.660 [95% CI 0.465–0.937, *p* = 0.020]. Analysis of EPA and DHA as continuous variables showed a modest negative association with MASLD risk (EPA: OR = 0.998, 95% CI 0.996–0.999, *p* = 0.037; DHA: OR = 0.999, 95% CI 0.998–1.000, *p* = 0.053), suggesting that higher omega-3 intake from Group 2 fish may slightly reduce the likelihood of developing MASLD.

Table 7 presents logistic regression results examining the association between MASLD and EPA and DHA intake from Group 3, analyzed as both continuous and categorical variables. In the multivariate analysis (Model 2), no statistically significant associations were observed for EPA or DHA intake from Group 3. Although the ORs were slightly below 1, the lack of statistical significance prevents drawing definitive conclusions.

## 4. Discussion

In this study, we investigated the association between omega-3 PUFAs, specifically EPA and DHA, and MASLD in a Mediterranean cohort from the NUTRIHEP, a prospective study conducted in a Mediterranean population with traditionally high fish consumption. Initiated in 2005–2006 with a systematic random sample of adults from Putignano (BA), Italy, the cohort underwent its first follow-up between 2015 and 2018. For the present analysis, we employed a cross-sectional design focusing on follow-up data from 1426 participants.

Our findings suggest that a higher intake of these fatty acids from fish is associated with a lower likelihood of MASLD. The protective effect was most pronounced for Group 2 fish, such as sardines and salmon, while Group 1 fish showed a smaller yet significant benefit. The consumption of mollusks and crustaceans also contributed to overall omega-3 intake. Although mollusks and crustaceans are a characteristic element of the food culture in this area, they are not consumed on a weekly basis or, in any case, less frequently than other types of fish. Importantly, participants with MASLD reported lower average intakes of EPA and DHA than those without, underscoring a potential protective role of these nutrients in liver health. While omega-3 fatty acids have been studied in NAFLD, evidence on MASLD remains limited. Previous research shows that low omega-3 and high omega-6 intake contribute to NAFLD development, whereas higher fish consumption reduces risk independently of age, sex, BMI, or caloric intake [47]. Rui Zhen Wang et al. reported that consuming ≥3 servings of fatty fish per week, compared with none, was associated with a 64% lower likelihood of NAFLD [48]. Mechanistically, EPA and DHA improve hepatic lipid metabolism by downregulating SREBP-1c, activating PPAR-α, and enhancing fatty acid β-oxidation, thereby reducing triglyceride accumulation [11,18,31,49]. Insufficient omega-3 intake impairs these pathways, favoring steatosis [33,50,51].

Beyond lipid metabolism, omega-3 PUFAs exert anti-inflammatory and antioxidant effects [52,53,54,55]. EPA and DHA lower pro-inflammatory cytokines, generate specialized pro-resolving mediators (resolvins and protectins), and reduce NF-κB activation through membrane-mediated signaling [56,57,58]. They also limit substrate availability for pro-inflammatory eicosanoids and scavenge reactive oxygen species [36,38]. These combined effects may protect against necroinflammation and oxidative injury in the liver.

The benefits of omega-3 fatty acids depend on maintaining a balanced dietary omega-6/omega-3 ratio, which is often skewed in Western diets. Excess omega-6 intake has been associated with NAFLD onset and progression to NASH [59]. In Western populations, linoleic acid intake (omega-6) is estimated to be 5–20 times higher than α-linolenic acid (omega-3) [11,18]. Low omega-3 and high omega-6 consumption may promote NAFLD development and, in affected individuals, accelerate progression to NASH [18,20,60]. Consuming fish 2–3 times per week helps restore this balance and supports liver health.

### Strengths and Limitations

The strengths of this study include its relatively large and well-characterized sample of 1297 adults, comprising both men and women, from a coastal Mediterranean region where access to and consumption of oily fish is common. This provides a unique opportunity to study the effects of omega-3 PUFAs in a population with high habitual intake. Another important strength is the focus on MASLD, a recently redefined and clinically relevant entity that has been less extensively studied compared to NAFLD. The diagnosis of MASLD in our cohort was established using standardized hepatic ultrasound with hepatic fat scored semi-quantitatively based on echotexture, echo penetration, intrahepatic vessel visibility, and diaphragm differentiation, which enhances the clinical robustness of our findings. Additionally, dietary intake was assessed with the validated EPIC Food Frequency Questionnaire (FFQ), which has been widely used in large-scale epidemiological studies. To minimize misclassification, the questionnaires were reviewed and cross-checked by trained nutritionists, thereby improving accuracy and reliability of the dietary data [40].

However, several limitations should be acknowledged. The cross-sectional nature of the study is its main limitation, as it prevents causal inference as with all FFQs, reliance on self-reported dietary intake introduces the possibility of recall bias and measurement error, which may lead to under- or overestimation of nutrient consumption. Our study did not include a direct measure of physical activity, an important lifestyle factor strongly associated with MASLD risk and progression, which could act as an unmeasured confounder [61]. Moreover, although we adjusted for multiple sociodemographic, dietary, and biochemical factors, the possibility of residual confounding cannot be excluded. Finally, environmental and cultural variability in fish consumption habits may limit the generalizability of our findings to populations with different dietary patterns [62].

## 5. Conclusions

In conclusion, our study suggests that higher intake of EPA and DHA from fish is associated with reduced risk of MASLD, particularly when derived from fatty fish rich in omega-3s. These findings support current dietary guidelines recommending regular fish consumption and highlight the potential role of omega-3 fatty acids in preventing or mitigating liver steatosis. A balanced diet including fish at least once per week can help achieve an appropriate omega-3 to omega-6 ratio [35], while recent guidelines suggest consuming fish 2–3 times per week, emphasizing variety across different types [63]. Stronger protective associations were observed for fatty fish with higher omega-3 content and bioavailability, whereas mollusks and crustaceans contributed less EPA and DHA per serving, yielding a smaller effect. No significant associations were found for some processed or frozen fish, likely due to lower omega-3 content or variable consumption patterns. Further longitudinal and interventional studies are needed to confirm causality and explore dose–response relationships [6,23].

## Figures and Tables

**Table 1:** Mean daily intake EPA and DHA (mg/day) from fish, as reported in the EPIC Food Frequency Questionnaire.

List of Fish	EPA (mg/day)	DHA (mg/day)
	Mean (SD)	Mean (SD)
Total Intake	139.78 (94.46)	242.39 (165.83)
**Group 1:**	16.91 (19.98)	12.22 (14.53)
Shrimps, Prawns, Langoustines	2.52 (3.93)	1.96 (3.06)
Octopus, Cuttlefish, Squid	10.71 (15.20)	7.92 (11.23)
Mussels, Clams	3.68 (5.41)	2.34 (3.44)
**Group 2:**	80.49 (72.34)	147.61 (131.16)
Sole, Plaice	4.79 (8.07)	5.99 (10.08)
Sardines, Mackerel, Anchovies	14.21 (25.61)	32.24 (58.09)
Trout	1.79 (4.51)	5.10 (12.88)
Salmon	30.08 (42.52)	40.22 (56.86)
Swordfish, Tuna	2.70 (5.09)	13.73 (25.90)
Other Fish	26.92 (38.18)	50.33 (71.38)
**Group 3:**	42.38 (36.95)	82.56 (80.79)
Salted cod, stockfish	3.69 (6.18)	6.24 (10.45)
Tinned fish	33.17 (32.48)	49.03 (48.01)
Frozen sticks and filets	5.52 (11.22)	27.28 (55.42)

EPA: eicosapentaenoic acid; DHA: docosahexaenoic acid. Tinned fish: tuna, mackerel, sardines, anchovies. Other fish: sea bream, sea bass, snapper.

**Table 2:** Participant characteristics by MASLD status, NUTRIHEP Study 2015–2018.

Variables ^a^		MASLD		
	Whole Sample ^b^	No	Yes	*p*-Value ^c^
N (%)	1297	668 (51.50)	629 (48.50)	
**Exposure variables**				
EPA (mg/day)	139.78 (94.46)	145.11 (92.28)	134.11 (96.48)	0.036
EPA Group 1 (mg/day)	16.91 (19.98)	17.31 (19.59)	16.50 (20.40)	0.470
EPA Group 2 (mg/day)	80.49 (72.34)	83.22 (70.03)	77.58 (74.67)	0.160
EPA Group 3 (mg/day)	42.38 (36.95)	44.59 (37.57)	40.03 (36.17)	0.027
DHA (mg/day)	242.39 (165.83)	251.24 (163.32)	232.99 (168.07)	0.048
DHA Group 1 (mg/day)	12.22 (14.53)	12.53 (14.35)	11.89 (14.72)	0.430
DHA Group 2 (mg/day)	147.61 (131.16)	151.16 (125.71)	143.84 (136.71)	0.320
DHA Group 3 (mg/day)	82.56 (80.79)	87.55 (84.03)	77.25 (76.93)	0.022
**Demographic and lifestyle characteristics**				
Age (years)	54.33 (14.34)	49.24 (13.80)	59.74 (12.86)	<0.001
Gender (%)				
Female	744 (57.40)	417 (56.00)	327 (44.00)	<0.001
Male	553 (42.60)	251 (45.40)	302 (54.60)	
rMED	8.04 (2.55)	7.91 (2.54)	8.18 (2.56)	0.050
rMED Score (%)				
Low	365 (28.10)	196 (53.70)	169 (46.30)	0.460
Moderate	705 (54.40)	362 (51.30)	343 (48.70)	
High	227 (17.50)	110 (48.50)	117 (51.50)	
Alcohol intake (g/day)	10.58 (12.72)	10.74 (13.41)	10.42 (11.96)	0.660
Wine intake (ml/day)	67.18 (174.36)	56.88 (214.44)	78.13 (116.89)	0.028
Kcal (day)	2056.26 (750.22)	2100.33 (724.88)	2009.46 (774.05)	0.029
Smoker (%)				
Never/Former	1137 (87.70)	587 (51.60)	550 (48.40)	0.870
Current	159 (12.30)	81 (50.90)	78 (49.10)	
Marital Status (%)				
Single	181 (14.00)	115 (63.50)	66 (36.50)	<0.001
Married or living together	1034 (79.70)	519 (50.20)	515 (49.80)	
Separated or divorced	28 (2.20)	20 (71.40)	8 (28.60)	
Widow/er	54 (4.20)	14 (25.90)	40 (74.10)	
Education (%)				
Primary school	282 (21.80)	71 (25.20)	211 (74.80)	<0.001
Secondary school	383 (29.50)	171 (44.60)	212 (55.50)	
High School	460 (35.50)	307 (66.70)	153 (33.30)	
Graduate	172 (13.30)	119 (69.20)	53 (30.80)	
Work (%)				
Managers and Professionals	102 (7.90)	57 (55.90)	45 (44.10)	<0.001
Craft, Agricultural, and Sales Workers	469 (36.20)	285 (60.80)	184 (39.20)	
Elementary Occupations	185 (14.10)	93 (50.30)	92 (49.70)	
Housewife	141 (10.90)	74 (52.50)	67 (47.50)	
Pensioners	325 (25.10)	110 (33.80)	215 (66.20)	
Unemployed	75 (5.80)	49 (65.30)	26 (34.70)	
Family income assessment (%)				
insufficient	27 (2.10)	10 (37.00)	17 (63.00)	0.025
just sufficient	167 (12.90)	81 (48.50)	86 (51.50)	
sufficient	1019 (78.60)	521 (51.10)	498 (48.90)	
more than sufficient	64 (4.90)	44 (68.80)	20 (31.20)	
good	20 (1.50)	12 (60.00)	8 (40.00)	
**Anthropometric and clinical parameters**				
BMI (kg/m^2^)	27.58 (5.05)	25.04 (3.59)	30.28 (4.97)	<0.001
Weight (kg)	72.93 (14.87)	66.66 (12.02)	79.58 (14.73)	<0.001
Waist (cm)	90.45 (13.46)	83.04 (10.38)	98.32 (11.79)	<0.001
SBP (mmHg)	120.93 (15.81)	115.64 (15.35)	126.52 (14.30)	<0.001
DBP (mmHg)	77.68 (8.00)	75.69 (7.88)	79.78 (7.58)	<0.001
Hypertension (%)				
No	847 (68.80)	517 (61.00)	330 (39.00)	<0.001
Yes	385 (31.20)	115 (29.90)	270 (70.10)	
Dyslipidemia (%)				
No	1047 (85.10)	561 (53.60)	486 (46.40)	<0.001
Yes	184 (14.90)	71 (38.60)	113 (61.40)	
Diabetes (%)				
No	1148 (93.20)	620 (54.00)	528 (46.00)	<0.001
Yes	84 (6.80)	12 (14.30)	72 (85.70)	
**Blood Tests**				
HbA1c (mmol/mol)	38.07 (6.87)	36.59 (5.05)	39.64 (8.09)	<0.001
Glucose (mg/dL)	95.34 (17.34)	90.13 (10.54)	100.89 (21.06)	<0.001
HOMA-IR	1.89 (1.88)	1.33 (0.90)	2.43 (2.38)	<0.001
ALT (U/L)	22.20 (16.21)	19.70 (8.27)	24.86 (21.37)	<0.001
γGT (U/L)	17.58 (13.46)	14.80 (7.67)	20.54 (17.16)	<0.001
AST (U/L)	21.74 (10.87)	20.70 (5.94)	22.85 (14.29)	<0.001
ALP (U/L)	52.98 (16.10)	50.10 (15.56)	56.04 (16.11)	<0.001
TG (mg/dL)	98.41 (69.23)	80.73 (58.55)	117.22 (74.60)	<0.001
TC (mg/dL)	191.35 (35.36)	188.90 (33.06)	193.96 (37.50)	0.010
HDL-C (mg/dL)	50.79 (12.59)	53.18 (12.80)	48.24 (11.85)	<0.001
C-reactive protein (mg/dL)	0.26 (0.55)	0.21 (0.52)	0.31 (0.58)	<0.001

Notes: ^a^ Values are expressed as means ± standard deviations. ^b^ Percentages are calculated by column; otherwise, percentages are calculated by row. ^c^ Continuous variables were compared using the Wilcoxon rank-sum test, and categorical variables using the χ^2^ test. Group 1: shrimps, prawns, langoustines, octopus, cuttlefish, squid, mussels, clams. Group 2: sole, plaice, sardines, mackerel, anchovies, trout, salmon, swordfish, tuna, other fish. Group 3: salted cod, stockfish, tinned fish, frozen sticks and filets. **Abbreviations:** MASLD: Metabolic Dysfunction-Associated Steatotic Liver Disease; EPA: eicosapentaenoic acid; DHA: docosahexaenoic acid; rMED: Relative Mediterranean Diet; BMI: body mass index; SBP: systolic blood pressure; DBP: diastolic blood pressure; HbA1c: glycosylated hemoglobin; HOMA: Homeostasis Model Assessment; ALT: alanine aminotransferase; γGT: gamma-glutamyl transferase; AST: aspartate aminotransferase; TG: triglycerides; TC: total cholesterol; HDL-C: high-density lipoprotein cholesterol; ALP: alkaline phosphatase.

**Table 3:** EPA and DHA intake from various fish species, categorized by MASLD status.

Variables	EPA (mg/day)			DHA (mg/day)		
	MASLD			MASLD		
	No	Yes		No	Yes	
	Mean (SD)	Mean (SD)	*p*-Value ^a^	Mean (SD)	Mean (SD)	*p*-Value ^a^
**Group 1:**	17.31 (19.59)	16.50 (20.40)	0.470	12.53 (14.35)	11.89 (14.72)	0.430
Shrimps, prawns, langoustines	2.64 (3.92)	2.40 (3.95)	0.270	2.05 (3.05)	1.87 (3.07)	0.270
Octopus, Cuttlefish, Squid	11.14 (16.12)	10.26 (14.15)	0.300	8.23 (11.92)	7.58 (10.46)	0.300
Mussels, Clams	3.53 (4.53)	3.84 (6.21)	0.310	2.25 (2.88)	2.44 (3.95)	0.310
**Group 2:**	83.22 (70.03)	77.58 (74.67)	0.160	151.16 (125.71)	143.84 (136.71)	0.320
Sole, Plaice	5.01 (8.41)	4.56 (7.69)	0.320	6.26 (10.51)	5.70 (9.62)	0.320
Sardines, Mackerel, Anchovies	12.43 (21.70)	16.11 (29.10)	0.010	28.20 (49.22)	36.53 (66.00)	0.010
Trout	2.04 (5.00)	1.51 (3.90)	0.034	5.84 (14.29)	4.32 (11.14)	0.034
Salmon	33.90 (45.70)	26.02 (38.49)	<0.001	45.33 (61.10)	34.79 (51.46)	<0.001
Swordfish, Tuna	3.03 (5.93)	2.35 (3.98)	0.016	15.41 (30.19)	11.95 (20.25)	0.016
Other Fish	26.81 (34.87)	27.04 (41.43)	0.910	50.12 (65.20)	50.55 (77.46)	0.910
**Group 3:**	44.59 (37.57)	40.03 (36.17)	0.027	87.55 (84.03)	77.25 (76.93)	0.022
Salted cod, stockfish	3.15 (5.78)	4.26 (6.53)	0.001	5.33 (9.79)	7.21 (11.04)	0.001
Tinned fish	35.38 (32.82)	30.81 (31.97)	0.011	52.31 (48.52)	45.55 (47.26)	0.011
Frozen sticks and filets	6.05 (11.95)	4.96 (10.36)	0.078	29.92 (59.03)	24.49 (51.21)	0.078

Notes: ^a^ Continuous variables were compared using the Wilcoxon rank-sum test. **Tinned fish:** tuna, mackerel, sardines, anchovies. **Other fish:** sea bream, sea bass, snapper. **Abbreviations:** MASLD: Metabolic Dysfunction-Associated Steatotic Liver Disease; EPA: eicosapentaenoic acid; DHA: docosahexaenoic acid.

**Table 4:** Logistic regression analysis of the association between total EPA and DHA intake and MASLD.

		Model 1			Model 2	
	OR ^a^	*p*-Value	95%CI	OR ^a^	*p*-Value	95%CI
EPA Quartiles (mg/day)						
<77	1.000			1.000		
77–119	0.686	0.017	0.503; 0.934	0.686	0.031	0.487; 0.966
120–182	0.712	0.031	0.523; 0.970	0.683	0.031	0.482; 0.966
183–630	0.613	0.002	0.450; 0.836	0.572	0.002	0.400; 0.818
						
Total EPA intake (mg/day)	0.999	0.037	0.998; 0.999	0.999	0.048	0.997; 0.999
	OR ^a^	*p*-value	95%CI	OR ^a^	*p*-value	95%CI
DHA Quartiles (mg/day)						
<130	1.000			1.000		
130–206	0.569	<0.001	0.417; 0.776	0.567	0.001	0.402; 0.799
207–318	0.738	0.054	0.542; 1.006	0.693	0.041	0.488; 0.985
319–950	0.554	<0.001	0.406; 0.757	0.516	<0.001	0.361; 0.739
						
Total DHA intake (mg/day)	0.999	0.048	0.998; 0.999	0.999	0.061	0.998; 1.000

Notes: EPA and DHA daily intake are presented both as continuous variables and by quartiles. ^a^ No MASLD: reference category. **Models:** Model 1: univariate; Model 2: adjusted for age (<40 vs. ≥40 years), sex, marital status, occupation, self-reported family income, rMED score, ALP, γGT, AST/ALT ratio, and C-reactive protein. **Abbreviations:** MASLD: Metabolic Dysfunction-Associated Steatotic Liver Disease; EPA: eicosapentaenoic acid; DHA: docosahexaenoic acid; rMED: Relative Mediterranean Diet; AST: aspartate aminotransferase; ALT: alanine aminotransferase; γGT: gamma-glutamyl transferase; ALP: alkaline phosphatase.

**Table 5:** Logistic regression analysis of the association between EPA and DHA intake from Group 1 intake and MASLD.

Group 1		Model 1			Model 2	
	OR ^a^	*p*-Value	95%CI	OR ^a^	*p*-Value	95%CI
EPA Quartiles (mg/day)						
<4.14	1.000			1.000		
4.14–10.79	0.817	0.196	0.600; 1.110	0.758	0.104	0.543; 1.058
11.80–23.84	0.673	0.012	0.494; 0.917	0.667	0.019	0.476; 0.936
23.85–198.0	0.752	0.071	0.552; 1.025	0.679	0.026	0.483; 0.955
						
Total EPA intake (mg/day)	0.998	0.467	0.993; 1.003	0.996	0.231	0.990; 1.002
	OR ^a^	*p*-value	95%CI	OR ^a^	*p*-value	95%CI
DHA Quartiles (mg/day)						
<2.80	1.000			1.000		
2.80–7.81	0.777	0.108	0.571; 1.057	0.709	0.044	0.507; 0.991
7.82–16.99	0.673	0.012	0.494; 0.917	0.647	0.012	0.461; 0.908
17.00–143.3	0.753	0.071	0.553; 1.025	0.684	0.029	0.487; 0.961
						
Total DHA intake (mg/day)	0.997	0.429	0.989; 1.005	0.995	0.217	0.986; 1.003

Notes: Daily EPA and DHA intake is presented both as continuous variables and by quartiles. ^a^ No MASLD: reference category. **Models:** Model 1: univariate; Model 2: adjusted for age (<40 vs. ≥40 years), sex, marital status, occupation, self-reported family income, rMED score, ALP, γGT, AST/ALT ratio, and C-reactive protein. Group 1: shrimps, prawns, langoustines, octopus, cuttlefish, squid, mussels, clams. **Abbreviations:** MASLD: Metabolic Dysfunction-Associated Steatotic Liver Disease; EPA: eicosapentaenoic acid; DHA: docosahexaenoic acid; rMED: Relative Mediterranean Diet; AST: aspartate aminotransferase; ALT: alanine aminotransferase; γGT: gamma-glutamyl transferase; ALP: alkaline phosphatase.

**Table 6:** Logistic regression analysis of the association between EPA and DHA intake from Group 2 and MASLD.

Group 2		Model 1			Model 2	
	OR ^a^	*p*-Value	95%CI	OR ^a^	*p*-Value	95%CI
EPA Quartiles (mg/day)						
<29.29	1.000			1.000		
29.30–64.00	0.890	0.456	0.654; 1.210	0.882	0.471	0.628; 1.240
64.01–110.2	0.816	0.195	0.599; 1.110	0.678	0.028	0.479; 0.960
110.3–470.0	0.677	0.013	0.497; 0.922	0.556	0.001	0.390; 0.794
						
Total EPA intake (mg/day)	0.999	0.161	0.997; 1.000	0.998	0.037	0.996; 0.999
	OR ^a^	*p*-value	95%CI	OR ^a^	*p*-value	95%CI
DHA Quartiles (mg/day)						
<54.6	1.000			1.000		
54.6–118.4	0.847	0.290	0.622; 1.152	0.832	0.289	0.592; 1.169
118.5–204.7	0.811	0.183	0.596; 1.104	0.660	0.020	0.465; 0.937
204.8–800.0	0.716	0.034	0.525; 0.975	0.575	0.002	0.403; 0.820
						
Total DHA intake (mg/day)	1.000	0.316	0.999; 1.000	0.999	0.053	0.998; 1.000

Notes: Daily EPA and DHA intake is shown both as continuous variables and by quartiles. ^a^ No MASLD: reference category. Models: Model 1: univariate; Model 2: adjusted for age (<40 vs. ≥40 years), sex, marital status, occupation, self-reported family income, rMED score, ALP, γGT, AST/ALT ratio, and C-reactive protein. Group 2: sole, plaice, sardines, mackerel, anchovies, trout, salmon, swordfish, tuna, other fish. Abbreviations: MASLD: Metabolic Dysfunction-Associated Steatotic Liver Disease; EPA: eicosapentaenoic acid; DHA: docosahexaenoic acid; rMED: Relative Mediterranean Diet; AST: aspartate aminotransferase; ALT: alanine aminotransferase; γGT: gamma-glutamyl transferase; ALP: alkaline phosphatase.

**Table 7:** Logistic regression analysis of the association between EPA and DHA intake from Group 3 and MASLD.

Group 3		Model 1			Model 2	
	OR ^a^	*p*-Value	95%CI	OR ^a^	*p*-Value	95%CI
EPA Quartiles (mg/day)						
<15.00	1.000			1.000		
15.00–34.67	0.777	0.108	0.571; 1.057	0.898	0.533	0.639; 1.261
34.68–59.00	0.744	0.060	0.547; 1.013	0.958	0.808	0.681; 1.349
59.00–280	0.682	0.015	0.501; 0.929	0.880	0.470	0.623; 1.244
						
Total EPA intake (mg/day)	0.997	0.027	0.994; 1.000	0.999	0.721	0.996; 1.003
	OR ^a^	*p*-value	95%CI	OR ^a^	*p*-value	95%CI
DHA Quartiles (mg/day)						
<25.50	1.000			1.000		
25.50–64.29	0.745	0.061	0.547; 1.014	0.816	0.241	0.582; 1.146
64.30–107.0	0.675	0.012	0.496; 0.918	0.871	0.430	0.617; 1.228
107.0–700.5	0.662	0.009	0.487; 0.902	0.857	0.381	0.607; 1.210
						
Total DHA intake (mg/day)	0.998	0.023	0.997; 1.000	1.000	0.620	0.998; 1.001

Notes: Daily EPA and DHA intake is presented both as continuous variables and by quartiles. ^a^ No MASLD: reference category. **Models:** Model 1: univariate; Model 2: adjusted for age (<40 vs. ≥40 years), sex, marital status, occupation, self-reported family income, rMED score, ALP, γGT, AST/ALT ratio, and C-reactive protein. Group 3: salted cod, stockfish, tinned fish, frozen sticks and filets. **Abbreviations:** MASLD: Metabolic Dysfunction-Associated Steatotic Liver Disease; EPA: eicosapentaenoic acid; DHA: docosahexaenoic acid; rMED: Relative Mediterranean Diet; AST: aspartate aminotransferase; ALT: alanine aminotransferase; γGT: gamma-glutamyl transferase; ALP: alkaline phosphatase.

## Data Availability

The data supporting the findings of this study are openly available at 10.6084/m9.figshare.30286522.

## Supplementary Materials

The following supporting information can be downloaded at https://www.mdpi.com/article/10.3390/nu17213372/s1, Figure S1: Ultrasound Scan Board.

## References

[1] Masarone M., Federico A., Abenavoli L., Loguercio C., Persico M. (2015). Non Alcoholic Fatty Liver: Epidemiology and Natural History. RRCT.

[2] Nobili V., Alisi A., Musso G., Scorletti E., Calder P.C., Byrne C.D. (2016). Omega-3 Fatty Acids: Mechanisms of Benefit and Therapeutic Effects in Pediatric and Adult NAFLD. Crit. Rev. Clin. Lab. Sci..

[3] Chan W.-K., Chuah K.-H., Rajaram R.B., Lim L.-L., Ratnasingam J., Vethakkan S.R. (2023). Metabolic Dysfunction-Associated Steatotic Liver Disease (MASLD): A State-of-the-Art Review. J. Obes. Metab. Syndr..

[4] Rinella M.E., Lazarus J.V., Ratziu V., Francque S.M., Sanyal A.J., Kanwal F., Romero D., Abdelmalek M.F., Anstee Q.M., Arab J.P. (2023). A Multisociety Delphi Consensus Statement on New Fatty Liver Disease Nomenclature. Hepatology.

[5] https://www.Progettopiter.It.

[6] Caldwell S. (2017). NASH Therapy: Omega 3 Supplementation, Vitamin E, Insulin Sensitizers and Statin Drugs. Clin. Mol. Hepatol..

[7] Byrne C.D., Targher G. (2015). NAFLD: A Multisystem Disease. J. Hepatol..

[8] De Wit N.J.W., Afman L.A., Mensink M., Müller M. (2012). Phenotyping the Effect of Diet on Non-Alcoholic Fatty Liver Disease. J. Hepatol..

[9] Dowman J.K., Armstrong M.J., Tomlinson J.W., Newsome P.N. (2011). Current Therapeutic Strategies in Non-Alcoholic Fatty Liver Disease. Diabetes Obes. Metab..

[10] Li W., Zheng L., Sheng C., Cheng X., Qing L., Qu S. (2011). Systematic Review on the Treatment of Pentoxifylline in Patients with Non-Alcoholic Fatty Liver Disease. Lipids Health Dis..

[11] Scorletti E., West A.L., Bhatia L., Hoile S.P., McCormick K.G., Burdge G.C., Lillycrop K.A., Clough G.F., Calder P.C., Byrne C.D. (2015). Treating Liver Fat and Serum Triglyceride Levels in NAFLD, Effects of PNPLA3 and TM6SF2 Genotypes: Results from the WELCOME Trial. J. Hepatol..

[12] Levene A.P., Goldin R.D. (2012). The Epidemiology, Pathogenesis and Histopathology of Fatty Liver Disease. Histopathology.

[13] Veena J., Muragundla A., Sidgiddi S., Subramaniam S. (2014). Non-Alcoholic Fatty Liver Disease: Need for a Balanced Nutritional Source. Br. J. Nutr..

[14] Softic S., Cohen D.E., Kahn C.R. (2016). Role of Dietary Fructose and Hepatic De Novo Lipogenesis in Fatty Liver Disease. Dig. Dis. Sci..

[15] Minno M.N.D.D. (2012). Omega-3 Fatty Acids for the Treatment of Non-Alcoholic Fatty Liver Disease. WJG.

[16] Mirmiran P., Hosseinpour-Niazi S., Naderi Z., Bahadoran Z., Sadeghi M., Azizi F. (2012). Association between Interaction and Ratio of ω-3 and ω-6 Polyunsaturated Fatty Acid and the Metabolic Syndrome in Adults. Nutrition.

[17] Simopoulos A.P. (2008). The Importance of the Omega-6/Omega-3 Fatty Acid Ratio in Cardiovascular Disease and Other Chronic Diseases. Exp. Biol. Med..

[18] Pachikian B.D., Essaghir A., Demoulin J.-B., Neyrinck A.M., Catry E., De Backer F.C., Dejeans N., Dewulf E.M., Sohet F.M., Portois L. (2011). Hepatic N-3 Polyunsaturated Fatty Acid Depletion Promotes Steatosis and Insulin Resistance in Mice: Genomic Analysis of Cellular Targets. PLoS ONE.

[19] Bouzianas D.G., Bouziana S.D., Hatzitolios A.I. (2013). Potential Treatment of Human Nonalcoholic Fatty Liver Disease with Long-Chain Omega-3 Polyunsaturated Fatty Acids. Nutr. Rev..

[20] Molendi-Coste O., Legry V., Leclercq I.A. (2011). Why and How Meet N-3 PUFA Dietary Recommendations?. Gastroenterol. Res. Pract..

[21] Rapoport S.I., Igarashi M., Gao F. (2010). Quantitative Contributions of Diet and Liver Synthesis to Docosahexaenoic Acid Homeostasis. Prostaglandins Leukot. Essent. Fat. Acids (PLEFA).

[22] Calder P.C. (2018). Very Long-Chain *n* -3 Fatty Acids and Human Health: Fact, Fiction and the Future. Proc. Nutr. Soc..

[23] Jump D.B., Lytle K.A., Depner C.M., Tripathy S. (2018). Omega-3 Polyunsaturated Fatty Acids as a Treatment Strategy for Nonalcoholic Fatty Liver Disease. Pharmacol. Ther..

[24] Janota B., Janion K., Buzek A., Janczewska E. (2025). Dietary Strategies in the Prevention of MASLD: A Comprehensive Review of Dietary Patterns Against Fatty Liver. Metabolites.

[25] Tan L.-J., Shin S. (2022). Effects of Oily Fish and Its Fatty Acid Intake on Non-Alcoholic Fatty Liver Disease Development among South Korean Adults. Front. Nutr..

[26] El-Badry A.M., Graf R., Clavien P.-A. (2007). Omega 3—Omega 6: What Is Right for the Liver?. J. Hepatol..

[27] Scorletti E., Byrne C.D. (2018). Omega-3 Fatty Acids and Non-Alcoholic Fatty Liver Disease: Evidence of Efficacy and Mechanism of Action. Mol. Asp. Med..

[28] Schaeffer L., Gohlke H., Müller M., Heid I.M., Palmer L.J., Kompauer I., Demmelmair H., Illig T., Koletzko B., Heinrich J. (2006). Common Genetic Variants of the FADS1 FADS2 Gene Cluster and Their Reconstructed Haplotypes Are Associated with the Fatty Acid Composition in Phospholipids. Hum. Mol. Genet..

[29] Shearer G.C., Savinova O.V., Harris W.S. (2012). Fish Oil—How Does It Reduce Plasma Triglycerides?. Biochim. Biophys. Acta (BBA)-Mol. Cell Biol. Lipids.

[30] Caviglia J.M., Gayet C., Ota T., Hernandez-Ono A., Conlon D.M., Jiang H., Fisher E.A., Ginsberg H.N. (2011). Different Fatty Acids Inhibit apoB100 Secretion by Different Pathways: Unique Roles for ER Stress, Ceramide, and Autophagy. J. Lipid Res..

[31] Takeuchi Y., Yahagi N., Izumida Y., Nishi M., Kubota M., Teraoka Y., Yamamoto T., Matsuzaka T., Nakagawa Y., Sekiya M. (2010). Polyunsaturated Fatty Acids Selectively Suppress Sterol Regulatory Element-Binding Protein-1 through Proteolytic Processing and Autoloop Regulatory Circuit. J. Biol. Chem..

[32] Khan S.A., Ali A., Khan S.A., Zahran S.A., Damanhouri G., Azhar E., Qadri I. (2014). Unraveling the Complex Relationship Triad between Lipids, Obesity, and Inflammation. Mediat. Inflamm..

[33] Stillwell W., Wassall S.R. (2003). Docosahexaenoic Acid: Membrane Properties of a Unique Fatty Acid. Chem. Phys. Lipids.

[34] Araya J., Rodrigo R., Pettinelli P., Araya A.V., Poniachik J., Videla L.A. (2010). Decreased Liver Fatty Acid Δ-6 and Δ-5 Desaturase Activity in Obese Patients. Obesity.

[35] Smit L.A., Mozaffarian D., Willett W. (2009). Review of Fat and Fatty Acid Requirements and Criteria for Developing Dietary Guidelines. Ann. Nutr. Metab..

[36] Cozzolongo R., Osella A.R., Elba S., Petruzzi J., Buongiorno G., Giannuzzi V., Leone G., Bonfiglio C., Lanzilotta E., Manghisi O.G. (2009). Epidemiology of HCV Infection in the General Population: A Survey in a Southern Italian Town. Am. J. Gastroenterol..

[37] Donghia R., Campanella A., Bonfiglio C., Cuccaro F., Tatoli R., Giannelli G. (2024). Protective Role of Lycopene in Subjects with Liver Disease: NUTRIHEP Study. Nutrients.

[38] Sever P. (2006). New Hypertension Guidelines from the National Institute for Health and Clinical Excellence and the British Hypertension Society. J. Renin Angiotensin Aldosterone Syst..

[39] Williams B., Mancia G., Spiering W., Agabiti Rosei E., Azizi M., Burnier M., Clement D., Coca A., De Simone G., Dominiczak A. (2018). 2018 Practice Guidelines for the Management of Arterial Hypertension of the European Society of Cardiology and the European Society of Hypertension: ESC/ESH Task Force for the Management of Arterial Hypertension. J. Hypertens..

[40] Pisani P. (1997). Relative Validity and Reproducibility of a Food Frequency Dietary Questionnaire for Use in the Italian EPIC Centres. Int. J. Epidemiol..

[41] Riboli E. (1997). The EPIC Project: Rationale and Study Design. European Prospective Investigation into Cancer and Nutrition. Int. J. Epidemiol..

[42] Hu H., Nakagawa T., Honda T., Yamamoto S., Mizoue T. (2024). Should Insulin Resistance (HOMA-IR), Insulin Secretion (HOMA-β), and Visceral Fat Area Be Considered for Improving the Performance of Diabetes Risk Prediction Models. BMJ Open Diabetes Res. Care.

[43] Chiloiro M., Caruso M.G., Cisternino A.M., Inguaggiato R., Reddavide R., Bonfiglio C., Guerra V., Notarnicola M., De Michele G., Correale M. (2013). Ultrasound Evaluation and Correlates of Fatty Liver Disease: A Population Study in a Mediterranean Area. Metab. Syndr. Relat. Disord..

[44] Simon S.D. (2001). Understanding the Odds Ratio and the Relative Risk. J. Androl..

[45] Ahrens A., Hansen C.B., Schaffer M.E. (2020). Lassopack: Model Selection and Prediction with Regularized Regression in Stata. Stata J..

[46] Kim J.H. (2019). Multicollinearity and Misleading Statistical Results. Korean J. Anesthesiol..

[47] Zelber-Sagi S., Nitzan-Kaluski D., Goldsmith R., Webb M., Blendis L., Halpern Z., Oren R. (2007). Long Term Nutritional Intake and the Risk for Non-Alcoholic Fatty Liver Disease (NAFLD): A Population Based Study. J. Hepatol..

[48] Wang R.Z., Zhang W.S., Jiang C.Q., Zhu F., Jin Y.L., Xu L. (2023). Association of Fish and Meat Consumption with Non-Alcoholic Fatty Liver Disease: Guangzhou Biobank Cohort Study. BMC Public Health.

[49] Jump D.B., Botolin D., Wang Y., Xu J., Demeure O., Christian B. (2008). Docosahexaenoic Acid (DHA) and Hepatic Gene Transcription. Chem. Phys. Lipids.

[50] Tanaka N., Zhang X., Sugiyama E., Kono H., Horiuchi A., Nakajima T., Kanbe H., Tanaka E., Gonzalez F.J., Aoyama T. (2010). Eicosapentaenoic Acid Improves Hepatic Steatosis Independent of PPARα Activation through Inhibition of SREBP-1 Maturation in Mice. Biochem. Pharmacol..

[51] Mehta S.R. (2010). Review: Advances in the Treatment of Nonalcoholic Fatty Liver Disease. Ther. Adv. Endocrinol..

[52] Calder P.C. (2013). Omega-3 Polyunsaturated Fatty Acids and Inflammatory Processes: Nutrition or Pharmacology?. Brit J. Clin. Pharmacol..

[53] Kajikawa S., Harada T., Kawashima A., Imada K., Mizuguchi K. (2010). Highly Purified Eicosapentaenoic Acid Ethyl Ester Prevents Development of Steatosis and Hepatic Fibrosis in Rats. Dig. Dis. Sci..

[54] Calder P.C. (2015). Marine Omega-3 Fatty Acids and Inflammatory Processes: Effects, Mechanisms and Clinical Relevance. Biochim. Biophys. Acta (BBA)-Mol. Cell Biol. Lipids.

[55] Valenzuela R., Espinosa A., González-Mañán D., D’Espessailles A., Fernández V., Videla L.A., Tapia G. (2012). N-3 Long-Chain Polyunsaturated Fatty Acid Supplementation Significantly Reduces Liver Oxidative Stress in High Fat Induced Steatosis. PLoS ONE.

[56] Huang T., Bhulaidok S., Cai Z., Xu T., Xu F., Wahlqvist M.L., Li D. (2010). Plasma Phospholipids *n* -3 Polyunsaturated Fatty Acid Is Associated with Metabolic Syndrome. Mol. Nutr. Food Res..

[57] Calder P.C. (2012). Long-Chain Fatty Acids and Inflammation. Proc. Nutr. Soc..

[58] Wong S.W., Kwon M.-J., Choi A.M.K., Kim H.-P., Nakahira K., Hwang D.H. (2009). Fatty Acids Modulate Toll-like Receptor 4 Activation through Regulation of Receptor Dimerization and Recruitment into Lipid Rafts in a Reactive Oxygen Species-Dependent Manner. J. Biol. Chem..

[59] Alvheim A.R., Malde M.K., Osei-Hyiaman D., Hong Y.H., Pawlosky R.J., Madsen L., Kristiansen K., Frøyland L., Hibbeln J.R. (2012). Dietary Linoleic Acid Elevates Endogenous 2-AG and Anandamide and Induces Obesity. Obesity.

[60] Takayama F., Nakamoto K., Totani N., Yamanushi T., Kabuto H., Kaneyuki T., Mankura M. (2010). Effects of Docosahexaenoic Acid in an Experimental Rat Model of Nonalcoholic Steatohepatitis. J. Oleo Sci..

[61] Franco I., Bianco A., Mirizzi A., Campanella A., Bonfiglio C., Sorino P., Notarnicola M., Tutino V., Cozzolongo R., Giannuzzi V. (2020). Physical Activity and Low Glycemic Index Mediterranean Diet: Main and Modification Effects on NAFLD Score. Results from a Randomized Clinical Trial. Nutrients.

[62] Fewell Z., Davey Smith G., Sterne J.A.C. (2007). The Impact of Residual and Unmeasured Confounding in Epidemiologic Studies: A Simulation Study. Am. J. Epidemiol..

[63] https://Sinu.It/Larn/.

