# Supplementary material for: Dietary Omega-3 Fatty Acids from Fish and Risk of Metabolic Dysfunction-Associated Steatotic Liver Disease in a Mediterranean Population: Findings from the NUTRIHEP Cohort
Source: Nutrients. 2025 Oct 27;17(21):3372. doi: 10.3390/nu17213372 (PMC12609867; doi:10.3390/nu17213372)
Supplement: Supplementary file 1 [file nutrients-17-03372-s001.zip › nutrients-3943468-supplementary.pdf]

Liver Ultrasound

**Dimensions**	Normal	Increase	Reduced
**Margins**	Regular	Irregulars	
**Ecostructure**	Homogeneous Normoechogenic	Uneven	Hyperechogenic

Evaluation of Hepatic Steatosis

**Contrast between liver parenchyma (P. EPA) and renal parenchyma (P. REN)**	**Deep beam penetration ultrasonic**	**Sharpness of the vascular structures, particularly particularly the veins**
Homogeneous echo level and contrast between P. EPA and P. REN not evident (0)	Hepatic parenchyma clearly visible from the surface to the diaphragm (0)	Vascular structures clearly visible (0)
Slight discrepancy in echogenicity hepatic-renal (1)	Presence of opacity of the deeper parts of the liver or failure to visualize the diaphragm (1)	Loss of echoes of the vascular structures (1)
Wide discrepancy between hepatic and renal (2)	Presenza di opacità delle parti più profonde del fegato e mancata visualizzazione del diaframma (2)	Vascular structures not clearly visible (2)
**Steatosis score**: Steatosis Absent (0); Mild Steatosis (1-2); Moderate Steatosis (3-5); Severe Steatosis (6)		
